# Lessons Learned From a Binational Survey to Examine Women’s Health Status in the US-Mexico Border Region

**Published:** 2008-09-15

**Authors:** Brian C. Castrucci, Evelyn Delgado, Vincent Fonseca, R.J. Dutton, Fouad Berrahou

**Affiliations:** Office of Title V, Division of Family and Community Health Services, Texas Department of State Health Services; Division of Family and Community Health Services, Texas Department of State Health Services, Austin, Texas; Texas Department of State Health Services, Austin, Texas; Office of Border Health, Texas Department of State Health Services, Austin, Texas; Office of Title V and Family Health, Division of Family and Community Health Services, Texas Department of State Health Services, Austin, Texas

## Introduction

The US-Mexico border region has experienced rapid population growth during the past 30 years. Conservative estimates suggest that the population of this region will increase by 34% between 2000 and 2030; more liberal estimates indicate a 97% increase (1). This population growth has been influenced in part by increased industrialization and trade, resulting from government policies in Mexico and the United States. An example of this increased trade is the more than 13,000 commercial freight crossings per day on the US-Mexico border, a 66% increase from 1999 (2).

As the economic and population transfer in the US-Mexico border region increases, improvements to health status of Texans living in this area require interventions and strategies based on binational collaboration. However, each country has its own policies and practices that shape health status. Understanding how these policies influence health behavior and the delivery of care on each side of the border creates opportunities to improve health status among women, infants, and children in both communities. To take advantage of these opportunities, standardized data on health beliefs and practices in this unique geographic region are needed.

Compared with other Texas residents, Texans living in the US-Mexico border region experience higher rates of communicable disease and self-described fair or poor health, lower rates of physical activity, higher obesity prevalence, and greater limitations to accessing and obtaining health insurance. This issue of *Preventing Chronic Disease* (*PCD*) explores challenges in maternal and reproductive health, using surveillance data collected through the Brownsville-Matamoros Sister City Project for Women's Health (BMSCP), funded in 2005 by the Centers for Disease Control and Prevention (CDC). Four of the articles in this issue of *PCD* address the time periods before pregnancy (3), during pregnancy (4,5), and after pregnancy (6). Analysis of the data presented in these articles creates an opportunity to understand the effect of different policies and practices on each side of the US-Mexico border, so each public health system can learn from the other and identify issues in which binational collaboration may be appropriate and necessary.

## Before Pregnancy: Family Planning Services

Texas has nearly 400 state-funded family planning clinics, including 11 in Cameron County. These clinics provide quality and comprehensive reproductive health care services that are low-cost and easily accessible. Family planning services promote the use of contraceptive methods that allow women to prevent, delay, space, or otherwise time pregnancies. Unlike women who have unintended pregnancies, women who plan their pregnancies may obtain appropriate preconception care, begin prenatal care earlier, and experience improved birth outcomes (7-10). According to data reported by Robles et al in this issue of *PCD*, nearly half of all women surveyed in Cameron County and Matamoros reported that their births were unintended or that they wanted to get pregnant later or not at all (3).

Decreasing unintended pregnancies is a common goal for public health leaders on each side of the US-Mexico border. Binational collaboration is needed to eliminate barriers that prevent women from accessing family planning services and to help them choose the most effective contraceptive methods (ie, intrauterine devices and contraceptive injections) or highly effective contraceptive methods (ie, pills and patch) that are easy to use consistently and correctly. However, about three-quarters of the women surveyed in Cameron County and Matamoros used the least effective methods of birth control (ie, foam, jelly, cream, condom, diaphragm, rhythm, or withdrawal) at first intercourse (3). Before the current unintended pregnancy, least effective methods of birth control were used by 52.0% of the women in Matamoros and by 36.6% of the women in Cameron County (3).

Reducing unintended pregnancy and promoting the adoption of the most effective methods of contraception are complex challenges that require education and messages tailored to the unique characteristics of the border population. Binational collaboration can ensure that messages and materials are consistent with the beliefs and practices of women on each side of the border and consider the demographic composition of the population living in the US-Mexico border region. The data presented by Robles et al provide valuable information on contraceptive use, population-level correlates of contraceptive use, and pregnancy patterns that can be used to create educational materials and messages needed to reduce the incidence of unintended pregnancy (3).

## During Pregnancy: HIV Testing and Cervical Cancer Screening

Health screenings are an important part of preventive care. They ensure that common, serious diseases are detected and treated, resulting in improved health status and outcomes and reductions in health care costs associated with more complex and invasive treatment of advanced disease. Limited access to health care is a barrier to receiving timely and appropriate health screening. The impact of limited access contributes to low rates of screening for HIV and cervical cancer in the US-Mexico border region. Women have increased access and opportunity to receive screenings during pregnancy. Geographic disparities in health screening rates can be reduced if women take advantage of these opportunities.

The lifetime HIV testing prevalence is 38.4% throughout Texas and 36.1% in the border region (11). However, as reported by Gossman et al in this issue of *PCD*, HIV screening rates among women who recently gave birth in Cameron County exceeded 90% (4). CDC guidance and Texas state policy contributed to achieving such a high prevalence of HIV testing. CDC recommends that HIV screening be included in the routine panel of prenatal screening tests for all pregnant women, unless the patient declines (12). Similarly, Texas requires prenatal care providers to notify women verbally at the first prenatal examination that an HIV test will be performed, unless the patient objects (13).

In Matamoros, the HIV screening rate during pregnancy was less than 60% (4). Although the HIV infection rate in Mexico among people aged 15 to 49 years is half the US rate (14), HIV prevalence is increasing in the US-Mexico border region among migrant workers and their partners (15-18). Loneliness, isolation, and depression among migrant workers while in the United States have led to increased risky sexual behavior and HIV infection (15-18). Migrant workers may acquire HIV infections in the United States and subsequently infect partners in Mexico (15-18). HIV screening during pregnancy is an opportunity to increase lifetime HIV screening in the population and help ensure positive birth outcomes. In 2007, Mexican policy related to HIV screening changed from screening only among women at high risk and women who tested positive for syphilis to screening among all pregnant women (with signed consent) as part of routine prenatal care. With migrant workers spending time on each side of the border, a coordinated binational approach to reducing incidence among migrant workers and preventing secondary transmission is needed. Outreach and education strategies that promote safe-sex practices and ensure that medical therapy is continued when away from home targeting migrant workers who are HIV-positive need to be developed and implemented binationally. This collaboration is a necessary part of a comprehensive strategy to reduce HIV incidence on each side of the US-Mexico border.

Prevalence of 3-year screening for cervical cancer was 80.2% for all Texas women and 68.2% for Texas women living in the US-Mexico border region (19). However, as reported by Castrucci et al in this issue of *PCD* (5), 3-year and lifetime cervical cancer screening rates among women who recently gave birth in Cameron County exceeded 90%, with the majority of women indicating that they received the test as part of prenatal care. Although ensuring Papanicolaou (Pap) testing among women who delivered a live infant capitalizes on an opportunity and contributes to increasing 3-year and lifetime Pap test prevalence, these data suggest that improved access may help reduce the disparity in Pap test prevalence between the border population and the remainder of Texas. In addition to being screened for cervical cancer during pregnancy, women up to 26 years of age who complete their postpartum visit should be counseled and offered vaccination against human papillomavirus, according to the guidance issued by the Advisory Committee on Immunization Practices (20).

## After Pregnancy: Attempted Breastfeeding

Early postpartum breastfeeding rates in Texas have met *Healthy People 2010* standards (21,22). However, according to Castrucci et al in this issue of *PCD*, rates in the US-Mexico border region are lower than those statewide (6). Improved breastfeeding rates can benefit the border region by decreasing health care costs, improving infant immunity, and reducing infant morbidity and mortality. Early postpartum feeding rates for Matamoros residents are higher than those for Cameron County residents and for Texas statewide (6). Although the Texas legislature has recognized breastfeeding as the best method of infant nutrition, clarified a woman's right to breastfeed in public, and established parameters for creating "mother-friendly" worksites, no regulations or requirements support breastfeeding in the hospital environment (23). Texas Ten Step is a voluntary program for hospitals that encourages birthing facilities to reach the goal of having 75% of their mothers breastfeeding at discharge; helps facilities support breastfeeding mothers before, during, and after delivery; and encourages facilities to identify breastfeeding resources for mothers after they are discharged (24). Although this program has improved the breastfeeding environment in participating hospitals, according to the 2006 Texas WIC (Special Supplemental Nutrition Program for Women, Infants, and Children) Infant Feeding Practices Survey, the proportion of respondents who reported receiving breastfeeding education from hospital staff and receiving advice to breastfeed on infant demand was lower in the border region than in the remainder of Texas (ie, about three-quarters of respondents reported receiving formula from the hospital) (25).

In contrast, Mexico's Ministry of Health has implemented clinical practice guidelines (*La Norma Oficial Mexicana*) that require exclusive breastfeeding to begin as soon as possible following delivery; support and facilitate breastfeeding on infant demand; set standards, criteria, and procedures that promote and protect exclusive breastfeeding; and require medical units to provide appropriate conditions for mothers to practice exclusive breastfeeding (26). Furthermore, policy in Mexico places restrictions on the distribution of formula in the hospital, restricting medical units from distributing or promoting free breast milk substitutes and employees from receiving incentives from the manufacturers of breast milk substitutes (26). The benefits of these policies are apparent in the higher rates of attempted breastfeeding in Matamoros compared with those in Cameron County (10).

Further study of clinical practice guidelines in Mexico and the implementation of these guidelines may help create future Texas policy and practices that promote breastfeeding. Public health leaders in Mexico could partner with local health authorities and hospital administrators in Texas to discuss possible barriers and solutions to implementing regulations that limit the distribution of infant formula.

## Conclusions

Confidentiality issues, resulting from legal and cultural restrictions, and differences in data collection and measurement practices, inhibit information sharing between Mexico and Texas (27). The BMSCP overcame these challenges. As part of this project, standardized data were collected on each side of the US-Mexico border. These data provide an opportunity for each health system to learn from the successes of the other and to identify opportunities for collaboration, with the goal of improving the public's health on each side of the US-Mexico border. However, the BMSCP data reported in this issue of *PCD* represent a single point in time and apply to only 1 of 14 pairs of sister cities on the US-Mexico border. Consistent and timely surveillance is needed to identify changes in established disease and behavior patterns, to understand new and emerging health threats, and to understand, evaluate, and document the effectiveness of collaborations. Expanding surveillance to other pairs of sister cities will increase our understanding of variations within and between pairs of sister cities and will provide insight into the health behaviors and trends throughout the border region.

The demand for policies, programs, and strategic initiatives to be built on sound epidemiologic information is increasing, and the BMSCP provided this foundation in maternal and reproductive health in a specific area of the border region at one point in time. If we are to improve the health of the people living in the US-Mexico border region, the need for increased, regular, and expanded surveillance is clear.
